# *Helicobacter Pylori* Promotes the Expression of Krüppel-Like Factor 5, a Mediator of Carcinogenesis, *In Vitro* and *In Vivo*

**DOI:** 10.1371/journal.pone.0054344

**Published:** 2013-01-23

**Authors:** Jennifer M. Noto, Tinatin Khizanishvili, Rupesh Chaturvedi, M. Blanca Piazuelo, Judith Romero-Gallo, Alberto G. Delgado, Shradha S. Khurana, Johanna C. Sierra, Uma S. Krishna, Giovanni Suarez, Anne E. Powell, James R. Goldenring, Robert J. Coffey, Vincent W. Yang, Pelayo Correa, Jason C. Mills, Keith T. Wilson, Richard M. Peek

**Affiliations:** 1 Department of Medicine, Division of Gastroenterology, Vanderbilt University Medical Center, Nashville, Tennessee, United States of America; 2 Departments of Medicine, Pathology and Immunology, and Developmental Biology, Washington University, St. Louis, Missouri, United States of America; 3 Departments of Medicine and Cell and Developmental Biology, Vanderbilt University Medical Center, Nashville, Tennessee, United States of America; 4 Departments of Surgery and Cell and Developmental Biology, Vanderbilt University Medical Center, Nashville, Tennessee, United States of America; 5 Department of Medicine, Stony Brook University, Stony Brook, New York, United States of America; 6 Department of Cancer Biology, Vanderbilt University Medical Center, Nashville, Tennessee, United States of America; 7 Department of Pathology, Microbiology, and Immunology, Vanderbilt University Medical Center, Nashville, Tennessee, United States of America; 8 Veterans Affairs Tennessee Valley Healthcare System, Nashville, Tennessee, United States of America; University of Hyderabad, India

## Abstract

*Helicobacter pylori* is the strongest known risk factor for the development of gastric adenocarcinoma. *H. pylori* expresses a repertoire of virulence factors that increase gastric cancer risk, including the *cag* pathogenicity island and the vacuolating cytotoxin (VacA). One host element that promotes carcinogenesis within the gastrointestinal tract is Krüppel-like factor 5 (KLF5), a transcription factor that mediates key cellular functions. To define the role of KLF5 within the context of *H. pylori*-induced inflammation and injury, human gastric epithelial cells were co-cultured with the wild-type *cag^+^ H. pylori* strain 60190. KLF5 expression was significantly upregulated following co-culture with *H. pylori*, but increased expression was independent of the *cag* island or VacA. To translate these findings into an *in vivo* model, C57BL/6 mice were challenged with the wild-type rodent-adapted *cag^+^ H. pylori* strain PMSS1 or a PMSS1 *cagE^−^* isogenic mutant. Similar to findings *in vitro*, KLF5 staining was significantly enhanced in gastric epithelium of *H. pylori*-infected compared to uninfected mice and this was independent of the *cag* island. Flow cytometry revealed that the majority of KLF5^+^ cells also stained positively for the stem cell marker, Lrig1, and KLF5^+^/Lrig1^+^ cells were significantly increased in *H. pylori*-infected versus uninfected tissue. To extend these results into the natural niche of this pathogen, levels of KLF5 expression were assessed in human gastric biopsies isolated from patients with or without premalignant lesions. Levels of KLF5 expression increased in parallel with advancing stages of neoplastic progression, being significantly elevated in gastritis, intestinal metaplasia, and dysplasia compared to normal gastric tissue. These results indicate that *H. pylori* induces expression of KLF5 in gastric epithelial cells *in vitro* and *in vivo*, and that the degree of KLF5 expression parallels the severity of premalignant lesions in human gastric carcinogenesis.

## Introduction

Gastric adenocarcinoma is the second most common cause of cancer-related death worldwide [1]. The strongest known risk factor for this malignancy is infection with the bacterial pathogen, *Helicobacter pylori*; however, only a fraction of colonized individuals ever develop cancer [2]. Gastric cancer risk is modified by interactions between *H. pylori* virulence factors and host cell constituents. The *H. pylori cag* pathogenicity island is a strain-specific virulence locus that encodes a bacterial type IV secretion system, which translocates the microbial effector protein CagA into host epithelial cells. Within host cells, CagA can induce cellular alterations that decrease the threshold for carcinogenesis, including proliferation and migration [3]. CagE is an essential component of the *cag* type IV secretion system and, based on homology, functions as an ATPase; loss of CagE leads to incomplete assembly of the secretion apparatus. The *cag* secretion system can also deliver peptidoglycan, a component of the bacterial cell wall, into host cells, further augmenting proinflammatory and mitogenic responses [2]. VacA is an independent *H. pylori* virulence factor that functions as a cytotoxin to increase cellular permeability and vacuolation [2].

A host factor that promotes carcinogenesis within the gastrointestinal tract is Krüppel-like factor 5 (*KLF5* in humans, *Klf5* in mice), a member of a family of zinc-finger transcription factors that possess highly conserved carboxy-terminal DNA-binding domains [4], [5], [6]. KLF5 regulates proliferation, differentiation, and apoptosis, and its expression is upregulated during development and in certain disease states, such as cancer [6], [7], [8], [9]. In intestinal cells, KLF5 promotes tumor progression [10], [11], [12] and mediates intestinal epithelial cell hyperproliferation and regenerative responses in response to infection and chronic inflammation [13], [14], [15].

In cultured cells, human KLF5 can act as a molecular chaperone for β-catenin, promoting its nuclear localization and modifying its transcriptional activity [16]. Recently, McConnell and colleagues demonstrated that intestinal cell-specific deletion of *Klf5* in mice leads to impaired barrier function, inflammation, and a regenerative phenotype [14], [17]. Tissue-specific depletion of *Klf5* in the intestine also resulted in disruption of β-catenin signaling, as evidenced by reductions in the levels of β-catenin target genes in *Klf5*-deficient compared to wild-type mice. Previous work from our laboratory has demonstrated that *H. pylori* can activate β-catenin and induce its nuclear translocation [18]. Since *H. pylori* increases the risk for gastric cancer and KLF5 mediates oncogenic pathways in the gastrointestinal tract, the aim of this study was to define the role of KLF5 in *H. pylori-*induced gastric inflammation and injury.

## Materials and Methods

### Ethics statement

All research involving human samples has been approved by the Institutional Review Board (IRB) of Vanderbilt University Medical Center and all human clinical investigations have been conducted according to the principles expressed in the Declaration of Helsinki. All research involving animals has been conducted in strict accordance with the recommendations in the Guide for the Care and Use of Laboratory Animals of the National Institutes of Health and all animal work has been approved by the Institutional Animal Care and Use Committee (IACUC) of Vanderbilt University Medical Center.

### *H. pylori* strains and growth conditions

The wild-type c*ag^+^ H. pylori* strain 60190, or isogenic 60190 *cagE^−^* (*cag* secretion system ATPase), *cagA^−^* (*cag* secretion system effector protein), *slt^−^* (soluble lytic transglycosylase, which decreases peptidoglycan synthesis), or *vacA^−^* (vacuolating cytotoxin) mutants, and the wild-type rodent-adapted c*ag^+^ H. pylori* strain PMSS1 or a PMSS1 *cagE^−^* isogenic mutant were cultured on trypticase soy agar with 5% sheep blood agar plates (BD Biosciences) for *in vitro* passage, as previously described [19]. Isogenic mutants were also cultured on Brucella agar (BD Biosciences) plates containing 20 µg/ml kanamycin (Sigma) to confirm presence of the kanamycin antibiotic resistance cassette. *H. pylori* strains were then cultured in Brucella broth (BD Biosciences) supplemented with 10% fetal bovine serum (Atlanta Biologicals) for 16 to 18 hours at 37°C with 5% CO_2_.

### Gastric epithelial cells and co-culture with *H. pylori*

AGS human gastric epithelial cells (ATCC), isolated from the stomach of a patient with gastric adenocarcinoma, were grown in RPMI 1640 (Life Technologies) supplemented with 10% fetal bovine serum (Atlanta Biologicals), L-glutamine (2 mM, BD Biosciences), and HEPES buffer (1 mM, Cellgro) at 37°C with 5% CO_2_. Wild-type *H. pylori* strain 60190 or its isogenic mutants were co-cultured with gastric epithelial cells at a multiplicity of infection (MOI) of 100∶1. *H. pylori* was heat-killed (HK) by boiling at 100°C for 10 minutes, as previously described [19]. Co-cultures were also performed in a transwell (TW) co-culture system (Costar**®**, Corning) with pore size of 0.4 µM at an MOI of 200∶1. For some experiments, gastric epithelial cells were pretreated with the transcriptional inhibitor actinomycin D (Calbiochem) for 1 hour at a concentration of 1 µg/ml and then co-cultured with *H. pylori*, as previously described [20]. For experiments using purified *H. pylori* lipopolysaccharide (LPS), gastric epithelial cells were treated with physiologic concentrations of LPS (10 ng/ml and 100 ng/ml) for 2 hours.

### Quantitative real-time reverse transcriptase-polymerase chain reaction

AGS cells were co-cultured with *H. pylori* strain 60190 or its isogenic mutants at an MOI of 100∶1 for 0.5, 1, or 2 hours. AGS cells were treated with *H. pylori* LPS at 10 ng/ml or 100 ng/ml for 2 hours. RNA was isolated using the RNeasy**®** RNA isolation kit (Qiagen), according to the manufacturer's instructions. Reverse transcriptase PCR and quantitative real-time PCR (Applied Biosystems, 7300 Real-Time PCR System) were performed, according to the manufacturer's instructions. Levels of human *KLF5* mRNA expression (TaqMan**®**, Applied Biosystems) were standardized to levels of human *GAPDH* mRNA expression (TaqMan**®**, Applied Biosystems).

### Western blot analysis

AGS cells were co-cultured with *H. pylori* strain 60190 or its isogenic mutants at an MOI of 100∶1 for 2, 4, or 8 hours. Protein lysates were harvested using RIPA buffer (50 mM Tris, pH 7.2; 150 mM NaCl; 1% Triton X-100; and 0.1% SDS) containing protease (Roche) and phosphatase (Sigma) inhibitors and protein concentrations were determined by a bicinchoninic acid (BCA) assay (Pierce). Proteins (40 µg) were separated by SDS-PAGE and transferred (Bio-Rad) to polyvinylidene difluoride membranes (PVDF, Millipore). Human KLF5 protein expression was quantified using a rabbit polyclonal anti-KLF5 antibody (1∶1000, Millipore). KLF5 expression was standardized to glyceraldehyde-3-phosphate dehydrogenase (GAPDH) using a mouse polyclonal anti-GAPDH antibody (1∶5000, Millipore). Primary antibodies were detected using goat anti-rabbit or goat anti-mouse horseradish peroxidase (HRP)-conjugated secondary antibodies (1∶5000, Santa Cruz Biotechnology). Protein levels were visualized by Western Lightning Chemiluminescence Reagent Plus (PerkinElmer) according to the manufacturer's instructions and then quantified by densitometry using the ChemiGenius Gel Bio Imaging System (Syngene).

### Murine model of *H. pylori* infection

All animal studies were carried out in strict accordance with the recommendations in the Guide for the Care and Use of Laboratory Animals of the National Institutes of Health. Vanderbilt University Medical Center's Institutional Animal Care and Use Committee (IACUC) approved all protocols and all efforts were made to minimize animal suffering. Male C57BL/6 mice were purchased from Harlan Laboratories and housed in the Vanderbilt University Animal Care Facilities in a room with a 12-hour light-dark cycle at 21°C to 22°C. Mice were orogastrically challenged with Brucella broth, as an uninfected (UI) control, with the mouse-adapted wild-type *cag^+^ H. pylori* strain PMSS1, or with a PMSS1 *cagE^−^* isogenic mutant. Mice were euthanized at 24, 48, or 72 hours or 1, 4, or 8 weeks post-challenge and gastric tissue was harvested for quantitative culture, immunohistochemistry, and flow cytometry.

### *H. pylori* quantitative culture

To assess *H. pylori* colonization, one fourth of the stomach was harvested and homogenized in sterile phosphate-buffered saline (PBS, Cellgro). Following serial dilution, samples were plated on selective trypticase soy agar (BD Biosciences) plates with 5% sheep blood (Fisher Scientific) containing vancomycin (Sigma-Aldrich, 20 µg/ml), nalidixic acid (Sigma-Aldrich, 10 µg/ml), bacitracin (Calbiochem, 30 µg/ml), and amphotericin B (Sigma-Aldrich, 2 µg/ml) and were incubated at 37°C with 5% CO_2_ for 5–6 days for isolation of *H. pylori*. Colonization density was defined as log colony forming units per gram of gastric tissue (log CFU/g).

### Immunohistochemistry and analysis of murine gastric tissue

To assess *H. pylori*-induced inflammation, linear strips of gastric tissue, extending from the squamocolumnar junction through the proximal duodenum, were fixed in 10% neutral-buffered formalin (Azer Scientific), paraffin-embedded, and stained with hematoxylin and eosin (H&E). A single pathologist (MBP), blinded to treatment groups, scored indices of inflammation. Severity of acute and chronic inflammation was graded on a scale of 0 to 3 in both the gastric antrum and corpus, leading to a maximum combined score of 12, as previously described [21].

To assess KLF5 expression in murine gastric tissue, immunohistochemical (IHC) analysis was performed on deparaffinized gastric tissue sections using a murine rabbit polyclonal anti-KLF5 antibody (1∶50, Lifespan Biosciences). A single pathologist (MBP), blinded to treatment groups, scored cytoplasmic and nuclear epithelial KLF5 IHC staining separately in the entire length of the antral mucosa. The percentage of KLF5^+^ epithelial cells was assessed semi-quantitatively and the intensity of epithelial KLF5 staining was graded on a scale of 1–3 (weak, moderate, or strong). The KLF5 IHC score was determined by multiplying the KLF5 staining intensity by the percentage of positively stained cells, as previously described [22].

To further investigate the relationship between KLF5 expression and progenitor cell properties, we performed immunohistochemistry (IHC) for Ki67 and KLF5 to highlight the isthmal region where stem cells are known to be located. IHC analysis was performed on murine gastric tissue sections using a murine rabbit polyclonal anti-KLF5 antibody (1∶200, Santa Cruz), a murine rabbit polyclonal anti-Ki67 antibody (1∶200, Abcam), and Mayer's Hematoxylin (Vector Laboratories). ABC and DAB Peroxidase substrate kits (Vector Laboratories) were used for IHC detection. Serial sectioning was performed, sections were stained with either anti-KLF5 or anti-Ki67 antibodies and Mayer's Hematoxylin, and co-localization was assessed within gastric epithelium.

### Flow cytometry analysis of murine gastric tissue

Gastric epithelial cells were isolated from frozen murine gastric tissue using a dissociation and dispersion technique, as previously described [23]. Briefly, gastric tissue was treated with 10 µM DTT at room temperature for 30 minutes and then with 1.0 mM EDTA for 30 minutes at 4°C. Dispersed cells were filtered through a 70 µm filter (BD Falcon™) to isolate single cells. Cells were fixed and permeabilized with 0.1% paraformaldehyde (Fisher Scientific) and ice-cold methanol (Fisher Scientific). Cells were then incubated with a mouse monoclonal anti-pancytokeratin antibody conjugated with allophycocyanin (APC, 1∶100, BD Biosciences), a rabbit polyclonal anti-Lrig1 antibody (1∶200, [24]), and a murine goat polyclonal anti-KLF5 antibody (1∶50, Santa Cruz Biotechnology) at room temperature for 20 minutes. Cells were washed and stained with hamster anti-rabbit secondary antibody conjugated with fluorescein isothiocyanate (FITC, 1∶400, BD Biosciences) and donkey anti-goat secondary antibody conjugated with phycoerythrine (PE, 1∶200, BD Biosciences). Cells were acquired using a LSR II Flow Cytometer (BD Biosciences) and pancytokeratin-positive cells were analyzed for KLF5 and Lrig1 expression by using FlowJo (Tree Star Inc.).

### Immunohistochemistry on human gastric mucosa

The Institutional Review Board (IRB) of Louisiana State University Health Sciences Center, the Committees on Ethics of Universidad del Valle and Hospital Departamental de Nariño in Colombia, and the Institutional Review Board (IRB) of Vanderbilt University Medical Center approved this protocol. Gastric antrum biopsy samples from patients residing in a high gastric cancer risk region in the Colombian Andean mountains, who were enrolled in an ongoing prospective study designed to study mechanisms of *H. pylori* carcinogenesis [25], were used for immunohistochemistry.

Immunohistochemistry was performed on paraffin-embedded biopsy samples from patients without *H. pylori* infection and normal gastric mucosa and from *H. pylori*-infected patients with non-atrophic gastritis, intestinal metaplasia (IM), or gastric dysplasia. Tissue samples were deparaffinized and stained with a polyclonal anti-KLF5 antibody (1∶300, Lifespan Biosciences). A single pathologist (MBP) scored cytoplasmic and nuclear KLF5 IHC staining separately by assessing the percentage of KLF5^+^ epithelial cells semi-quantitatively.

### Statistical analysis

All experiments were performed on at least three independent occasions. Statistical analysis was performed using Student's t, Mann-Whitney, or ANOVA tests in GraphPad PRISM. A P value of <0.05 was considered statistically significant.

## Results

### *H. pylori* upregulates KLF5 in human gastric epithelial cells *in vitro*

**Figure 1:**
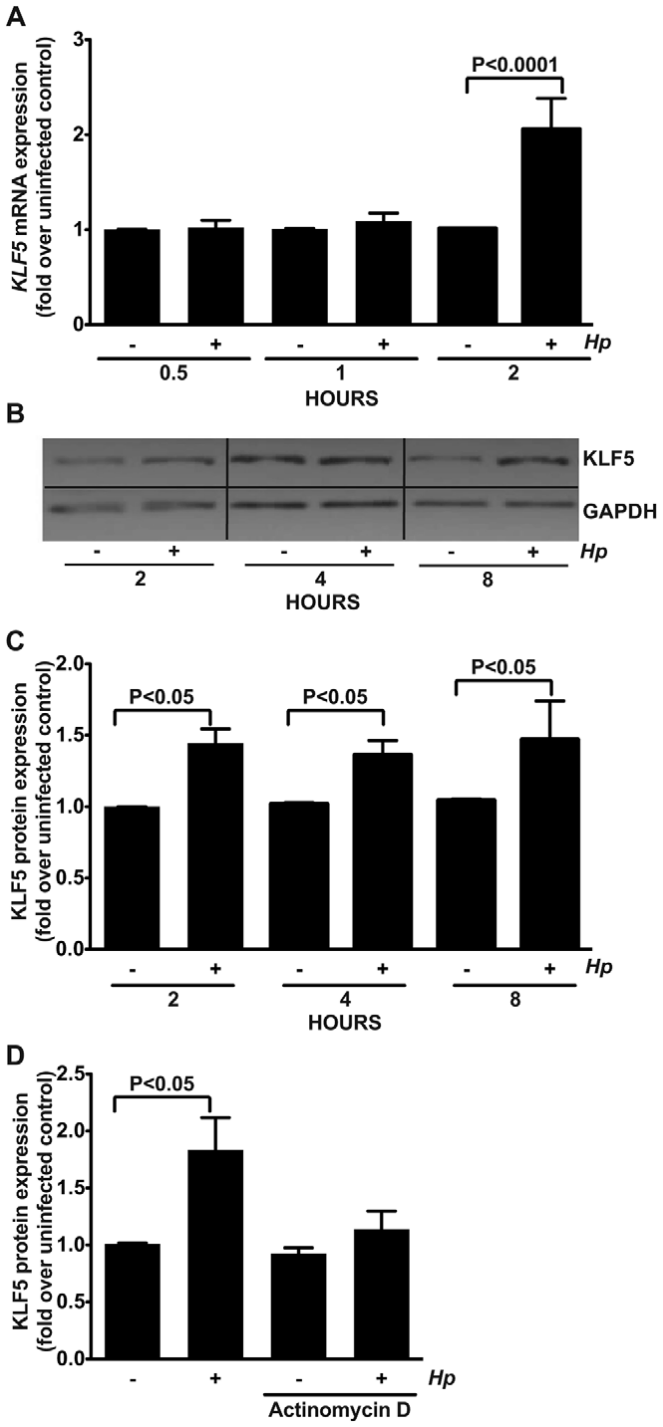
*H. pylori* upregulates KLF5 in human gastric epithelial cells *in vitro*. AGS human gastric epithelial cells were co-cultured with wild-type *cag^+^ H. pylori* strain 60190 at an MOI of 100∶1 for the indicated time points. (A) Quantitative real-time RT-PCR was used to assess *KLF5* mRNA expression relative to *GAPDH* mRNA expression. (B) Western blot analysis was used to assess KLF5 protein expression relative to GAPDH protein expression. (C) Western blot analysis replicates were quantified using densitometry. (D) Gastric epithelial cells were either left untreated or pretreated with actinomycin D for 1 hour prior to co-culture with *H. pylori*. Western blot analysis was used to assess KLF5 protein expression relative to GAPDH protein expression. Data are represented as fold over uninfected control. − and + symbols indicate the absence or presence of *H. pylori* (*Hp*), respectively. Error bars indicate standard error of the mean from experiments performed on at least three independent occasions, and Mann-Whitney tests were used to determine statistical significance between groups.

To define the effects of *H. pylori* on KLF5 expression, AGS human gastric epithelial cells were co-cultured with the wild-type *cag^+^ H. pylori* strain 60190 and levels of *KLF5* mRNA and KLF5 protein expression were determined by quantitative real-time RT-PCR (Figure 1A) and Western blot analysis (Figure 1B and 1C), respectively. *H. pylori* significantly increased *KLF5* mRNA expression two hours post-infection (Figure 1A). Concordant with increased levels of *KLF5* transcript, *H. pylori* significantly upregulated KLF5 protein expression, which peaked between two and eight hours post-infection (Figure 1B and 1C).

To determine whether *H. pylori*-induced upregulation of KLF5 is transcriptionally mediated, AGS human gastric epithelial cells were pretreated with the transcriptional inhibitor, actinomycin D, and then co-cultured with strain 60190 for two hours, an optimal time point for KLF5 induction (Figure 1A, 1B, and 1C). Actinomycin D pretreatment significantly attenuated *H. pylori*-induced KLF5 expression (Figure 1D), indicating that *H. pylori*-induced upregulation of KLF5 is transcriptionally mediated.

### *H. pylori*-induced KLF5 upregulation is independent of the *cag* pathogenicity island, VacA, or LPS

**Figure 2:**
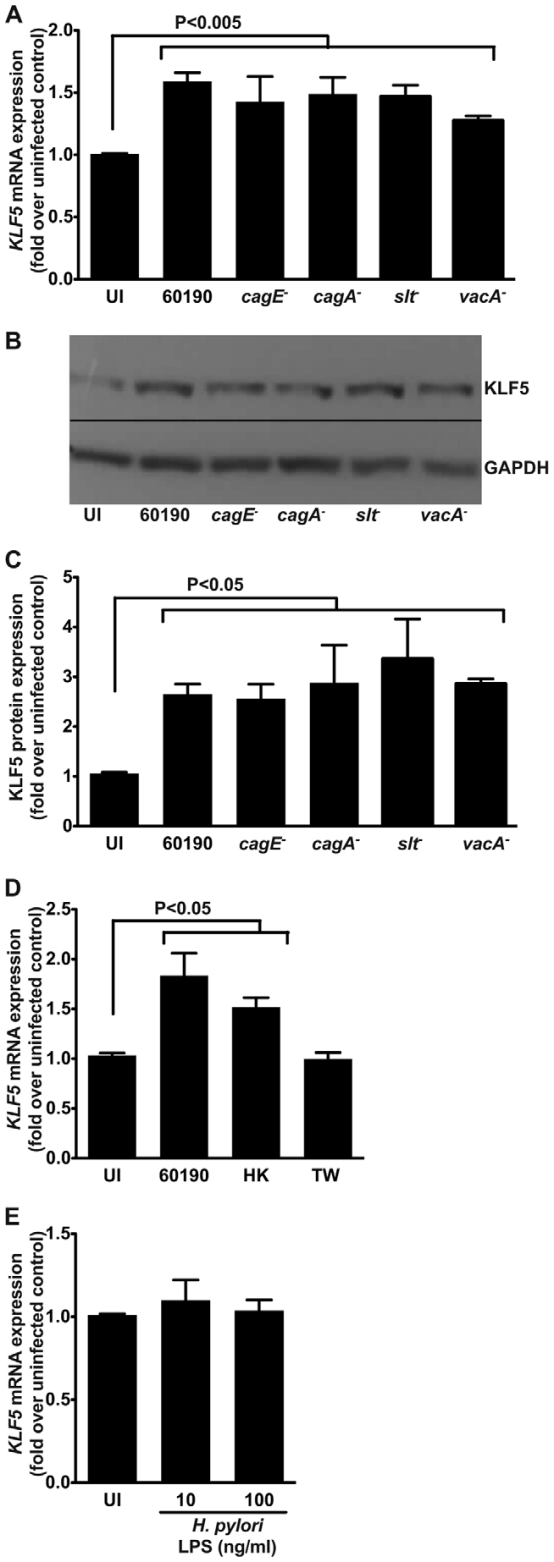
*H. pylori*-induced KLF5 upregulation is independent of the *cag* pathogenicity island, VacA, or LPS. AGS human gastric epithelial cells were co-cultured with wild-type *cag^+^ H. pylori* strain 60190, or its isogenic *cagE^−^*, *cagA^−^*, *slt^−^*, or *vacA^−^* mutants at an MOI of 100∶1 for 2 hours. (A) Quantitative real-time RT-PCR was used to assess *KLF5* mRNA expression relative to *GAPDH* mRNA expression. (B) Western blot analysis was used to assess KLF5 protein expression relative to GAPDH protein expression. (C) Western blot analysis replicates were quantified using densitometry. (D) Gastric epithelial cells were co-cultured with the wild-type *cag^+^ H. pylori* strain 60190, heat-killed (HK) *H. pylori* strain 60190, or with strain 60190 in a transwell (TW) system for 2 hours and quantitative real-time RT-PCR was used to assess *KLF5* mRNA expression relative to *GAPDH* mRNA expression. (E) Gastric epithelial cells were treated with *H. pylori* LPS (10 ng/ml or 100 ng/ml) for 2 hours and quantitative real-time RT-PCR was used to assess *KLF5* mRNA expression relative to *GAPDH* mRNA expression. Data are represented as fold over uninfected (UI) control. Error bars indicate standard error of the mean from experiments performed on at least three independent occasions, and Mann-Whitney tests were used to determine statistical significance between groups.

Several strain-specific microbial factors have been shown to mediate *H. pylori* pathogenesis. One virulence constituent is the *cag* pathogenicity island, which encodes a type IV secretion system that delivers the effector proteins, CagA or peptidoglycan, into host cells. To assess the role of the *cag* type IV secretion system and CagA, isogenic *cagE^−^* and *cagA^−^* mutants were utilized, respectively. To determine the role of peptidoglycan, a mutant lacking soluble lytic transglycosylase (*slt^−^*), which decreases peptidoglycan synthesis, was used. Another important *H. pylori* virulence factor is the vacuolating cytotoxin (VacA); thus, an isogenic *vacA^−^* mutant was also utilized. Consistent with the previous results (Figure 1), wild-type *H. pylori* strain 60190 induced significantly higher levels of *KLF5* mRNA (Figure 2A) and KLF5 protein (Figure 2B and 2C) compared to uninfected controls; however isogenic inactivation of *cagE^−^*, *cagA^−^*, *slt^−^*, or *vacA^−^* did not significantly affect this induction, indicating that these virulence factors are not required for *H. pylori*-induced upregulation of KLF5 (Figure 2A, 2B, and 2C).

To determine whether upregulation of KLF5 was dependent on live *H. pylori* or direct bacterial:host cell contact, gastric epithelial cells were co-cultured with heat-killed (HK) *H. pylori* or viable wild-type *H. pylori* added to a transwell (TW) system that separates bacteria from gastric epithelial cells, respectively (Figure 2D). Heat-killed *H. pylori*, but not viable *H. pylori* within the transwell, induced *KLF5* expression, similar to co-culture with the wild-type *H. pylori* strain 60190.

To assess whether the highly conserved *H. pylori* cell wall component, lipopolysaccharide (LPS), altered *KLF5* expression levels, gastric epithelial cells were treated with physiologic concentrations of purified *H. pylori* LPS. Treatment of cells with either 10 ng/ml or 100 ng/ml *H. pylori* LPS did not alter *KLF5* expression compared to uninfected controls (Figure 2E).

### *H. pylori* induces inflammation and KLF5 expression in a *cag*-independent manner within gastric epithelium *in vivo*

**Figure 3:**
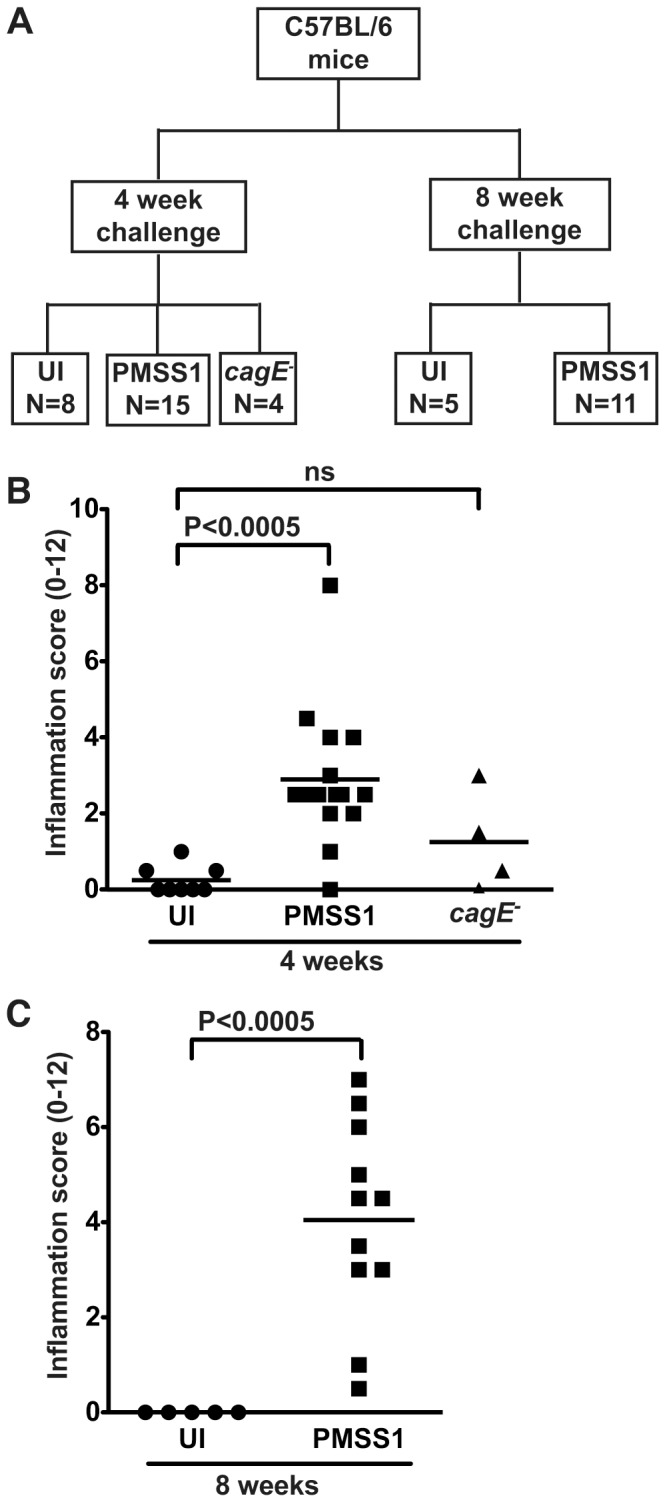
*H. pylori* induces inflammation in an *in vivo* C57BL/6 murine model. (A) C57BL/6 mice were challenged with Brucella broth, as an uninfected (UI) negative control, with the mouse-adapted wild-type *cag^+^ H. pylori* strain PMSS1, or a PMSS1 *cagE^−^* isogenic mutant for 4 or 8 weeks. (B and C) A single pathologist, blinded to treatment groups, assessed and scored inflammation at 4 weeks (B) and 8 weeks (C). Acute and chronic inflammation in both the antrum and corpus was scored on a scale of 0–3, leading to a possible maximum score of 12. Each data point represents an individual animal and mean values are shown. Circles designate uninfected mice, squares represent *H. pylori* PMSS1-infected mice, and triangles represent *H. pylori* PMSS1 *cagE^−^*-infected mice. Mann-Whitney and ANOVA tests were used to determine statistical significance between groups.

To extend our *in vitro* results into an *in vivo* model of *H. pylori* infection, C57BL/6 mice were challenged with Brucella broth as a negative uninfected (UI) control, wild-type mouse-adapted *cag^+^ H. pylori* strain PMSS1, or a PMSS1 *cagE^−^* isogenic mutant for 4 or 8 weeks (Figure 3A). Colonization efficiency, defined as the percentage of successfully colonized mice, was 100% for wild-type PMSS1 and the *cagE^−^* isogenic mutant at all time points (data not shown). As expected, infection with *H. pylori* strain PMSS1 resulted in significantly increased inflammation within the gastric mucosa compared to uninfected controls while levels of inflammation following infection with the *cagE^−^* isogenic mutant were no different than controls (Figure 3B and 3C).

**Figure 4:**
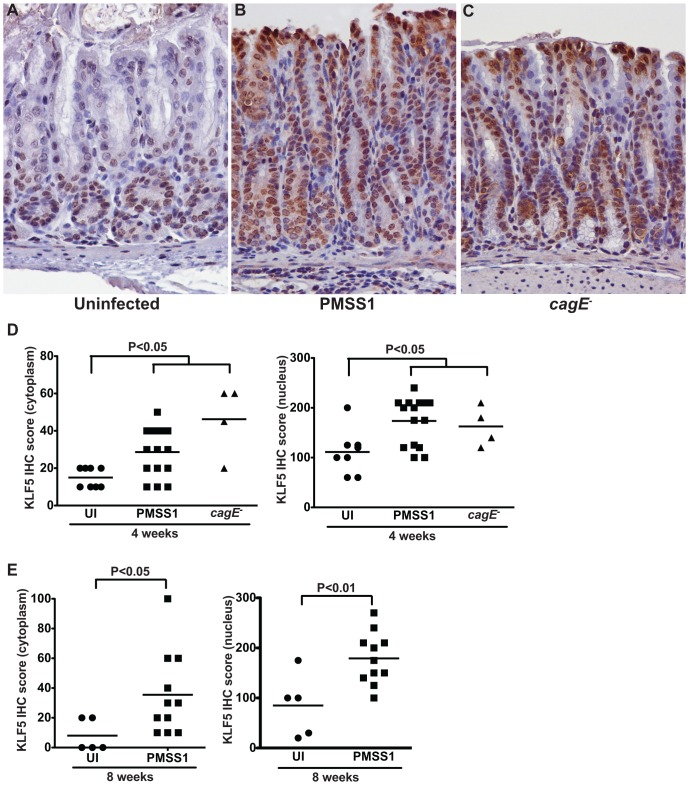
*H. pylori* upregulates KLF5 expression *in vivo*. (A–C) KLF5 expression in murine antral gastric tissue was assessed by KLF5 immunostaining in uninfected (A), *H. pylori* PMSS1-infected mice (B), and *H. pylori* PMSS1 *cagE^−^*-infected mice (C) at 400× magnification. (D and E) A single pathologist, blinded to treatment groups, assessed and scored KLF5 immunostaining. KLF5 immunohistochemistry (IHC) score was determined by assessing the percentage of KLF5^+^ epithelial cells multiplied by the intensity of epithelial KLF5 staining (1–3) in both the cytoplasm and nucleus of murine gastric epithelial cells (D and E). Each data point represents an individual animal and mean values are shown. Circles designate uninfected mice, squares represent *H. pylori* PMSS1-infected mice, and triangles represent *H. pylori* PMSS1 *cagE^−^*-infected mice. Mann-Whitney and ANOVA tests were used to determine statistical significance between groups.

To assess upregulation of KLF5 in this model, immunohistochemistry (IHC) was performed. KLF5 immunostaining was maximal within the gastric epithelial proliferative zone (isthmus zone) within the antrum of uninfected mice (Figure 4A). The intensity and magnitude of KLF5 immunostaining increased significantly in *H. pylori*-infected gastric epithelial cells, compared to uninfected gastric tissue, and expression expanded beyond the isthmal zone to include nearly all cells (Figure 4B, 4C, 4D, and 4E). Expression of KLF5 was predominantly nuclear, consistent with its function as a transcription factor; however, significant cytoplasmic staining was also evident in infected epithelial cells. Despite differences in levels of gastric inflammation in mice infected with PMSS1 versus the *cagE^−^* isogenic mutant (Figure 3B), both strains induced significantly increased levels of KLF5 compared to uninfected controls (Figure 4A–D). These findings are consistent with our *in vitro* data demonstrating that KLF5 is induced by *H. pylori* in a *cagE*-independent manner.

**Figure 5:**
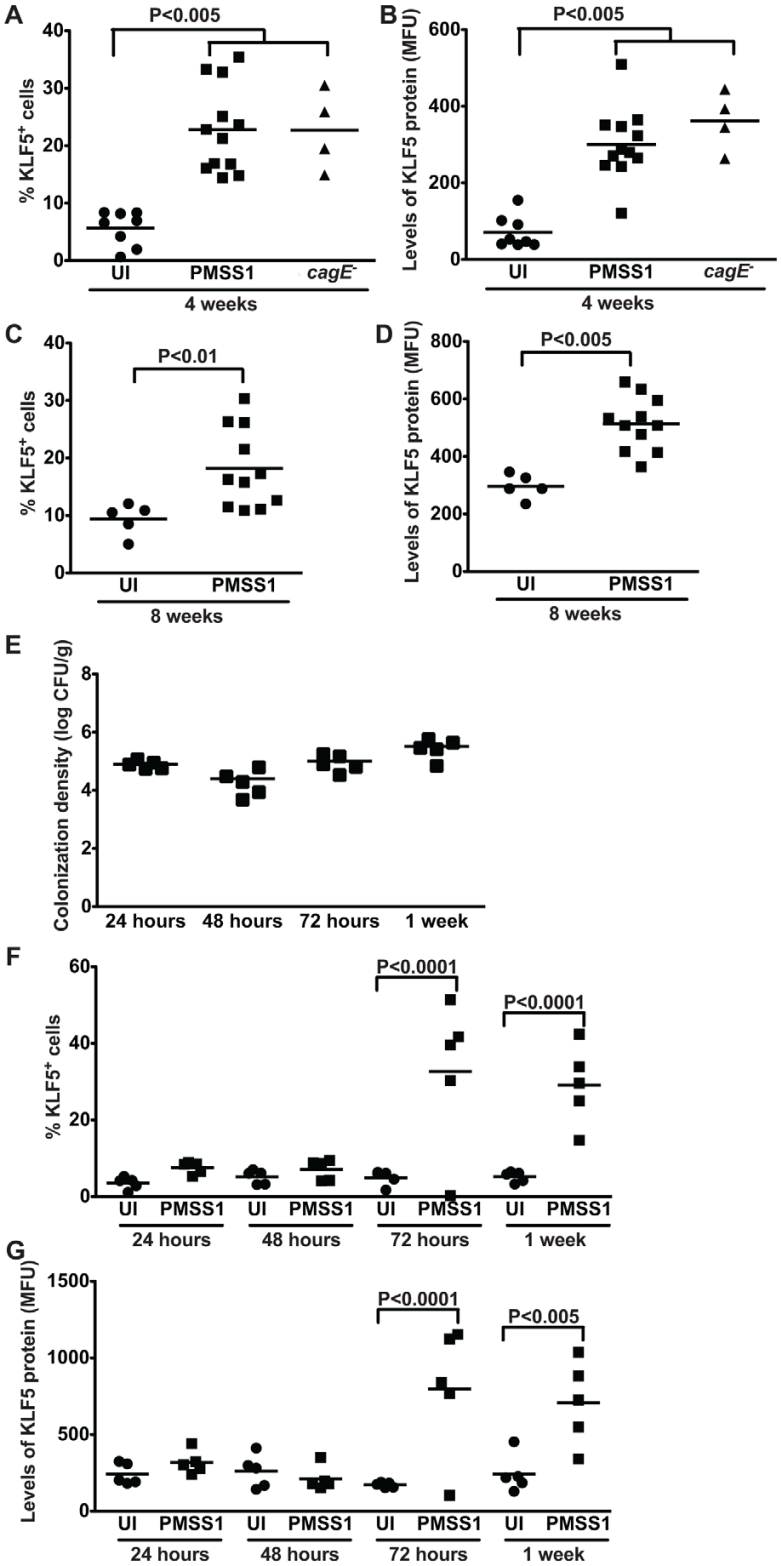
*H. pylori* induces expansion of a KLF5^+^ cell population *in vivo*. (A–G) KLF5 expression in murine gastric epithelial cells was assessed by flow cytometry analysis in uninfected and *H. pylori*-infected mice at acute time points (24, 48, 72 hours, and 1 week) and chronic time points (4 and 8 weeks) post-challenge. Percentage of KLF5^+^ cells at 4 weeks (A) and 8 weeks (C) and levels of KLF5 protein at 4 weeks (B) and 8 weeks (D), as determined by mean fluorescence units (MFU), were determined by flow cytometry. Data from 4 and 8 week time points were analyzed at separate times. *H. pylori* colonization density in mice infected for 24, 48, and 72 hours, and 1 week was assessed by quantitative culture (E). Percentage of KLF5^+^ cells (F) and levels of KLF5 protein (G) at 24, 48, or 72 hours, or 1 week were determined by flow cytometry. Each data point represents gastric epithelial cells analyzed from a single animal and mean values are shown. Circles designate uninfected mice, and squares represent *H. pylori*-infected mice. Mann-Whitney and ANOVA tests were used to determine statistical significance between groups.

We next examined KLF5 expression in epithelial cells isolated from uninfected and infected murine gastric tissue by flow cytometry analysis. Consistent with the KLF5 immunohistochemistry (Figure 4), flow cytometry demonstrated a significant increase in the percentage of KLF5^+^ cells in *H. pylori*-infected mice at both 4 and 8 weeks (Figure 5A and 5C). Levels of KLF5 protein, as determined by mean fluorescent units (MFU), were also significantly increased in *H. pylori*-infected mice compared to uninfected controls (Figure 5B and 5D). Consistent with the immunohistochemistry results, infection with wild-type strain PMSS1 or the PMSS1 *cagE^−^* isogenic mutant induced similar increases in the percentage of KLF5^+^ cells and levels of KLF5 protein (Figure 5A and 5B), confirming that induction of KLF5 occurs in a *cagE*-independent manner.

To determine if KLF5 upregulation was mediated by the host inflammatory response or by the effects of *H. pylori per se*, we assessed the expression of KLF5 during acute *H. pylori* infection *in vivo*. C57BL/6 mice were challenged with Brucella broth as a negative uninfected (UI) control or *H. pylori* strain PMSS1 for 24, 48, or 72 hours, or 1 week. Colonization efficiency was 100%, colonization density was similar at all time points (Figure 5E), and there was no evidence of inflammation (data not shown). Expression of KLF5 in gastric epithelial cells from uninfected and infected mice was evaluated by flow cytometry, which demonstrated a significant increase in the percentage of KLF5^+^ cells in *H. pylori*-infected mice at 72 hours and 1 week post-infection (Figure 5F), prior to the development of inflammation. Levels of KLF5 protein, as determined by mean fluorescent units (MFU), were also significantly increased in *H. pylori*-infected mice compared to uninfected controls at these times points (Figure 5G). Collectively, these data suggest that *H. pylori* upregulates KLF5 during both acute and chronic periods of colonization *in vivo*.

### *H. pylori* induces expansion of a KLF5-positive cell population that is also positive for the stem cell marker, Lrig1

**Figure 6:**
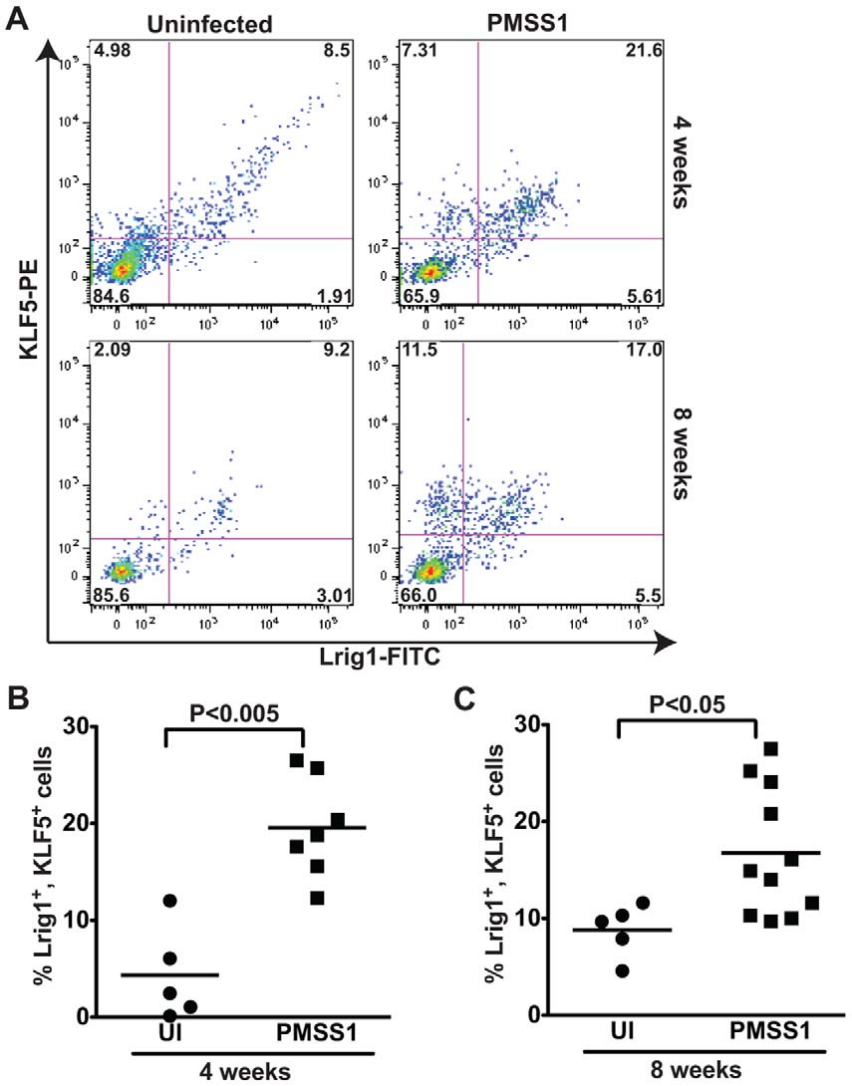
*H. pylori* induces expansion of a KLF5^+^, Lrig1^+^ cell population *in vivo*. (A) Flow cytometry dot plots demonstrate Lrig1 and KLF5 immunostaining in representative gastric epithelial cells from uninfected and *H. pylori*-infected mice at 4 and 8 weeks. The percentage of Lrig1^+^, KLF5^+^ cells was quantified in uninfected and *H. pylori*-infected mice at 4 weeks (B) and 8 weeks (C). Each data point represents gastric epithelial cells analyzed from a single animal and mean values are shown. Circles designate uninfected mice, and squares represent *H. pylori*-infected mice. Mann-Whitney and ANOVA tests were used to determine statistical significance between groups.

Infection with *H. pylori* can lead to atrophic gastritis and an expansion of stem and progenitor cell activity. Given that KLF5 is normally expressed in the progenitor zone of the gastric gland, and that its expression expands in response to *H. pylori*, we hypothesized that KLF5 expression might mark a progenitor population. Few reports exist regarding specific gastric epithelial progenitor markers and most of these are limited based on suboptimal specificity for progenitor cells or techniques that used promoter expression and not flow cytometry or immunohistochemistry [26]. Therefore, we decided to test whether KLF5-positive cells co-expressed a new marker of stem cells, Lrig1 (Leucine-rich Repeats and ImmunoGlobulin-like domains). Lrig1 marks non-cycling quiescent stem cells at the intestinal crypt bases and regulates repair following tissue damage [24], [27]. Lrig1 is expressed in gastric epithelium [28], though its cell specificity has not been previously assessed. When murine gastric epithelial cells were assessed for both KLF5 and Lrig1 expression by flow cytometry, a population of cells that were positively stained for both markers was observed. At 4 weeks, 75% of KLF5^+^ cells from infected mice were also Lrig1^+^ and conversely, 80% of Lrig1^+^ cells were KLF5^+^. At 8 weeks a similar trend was observed, whereby 60% of KLF5^+^ cells from infected mice were Lrig1^+^ and 76% of Lrig1^+^ cells were KLF5^+^ (Figure 6A). This population of KLF5^+^, Lrig1^+^ cells increased significantly in *H. pylori*-infected compared to uninfected tissues at both 4 weeks (Figure 6B) and 8 weeks (Figure 6C) post-infection. These results suggest that infection with *H. pylori* results in upregulation of KLF5 and expansion of a KLF5^+^, Lrig1^+^ cell population *in vivo*.

**Figure 7:**
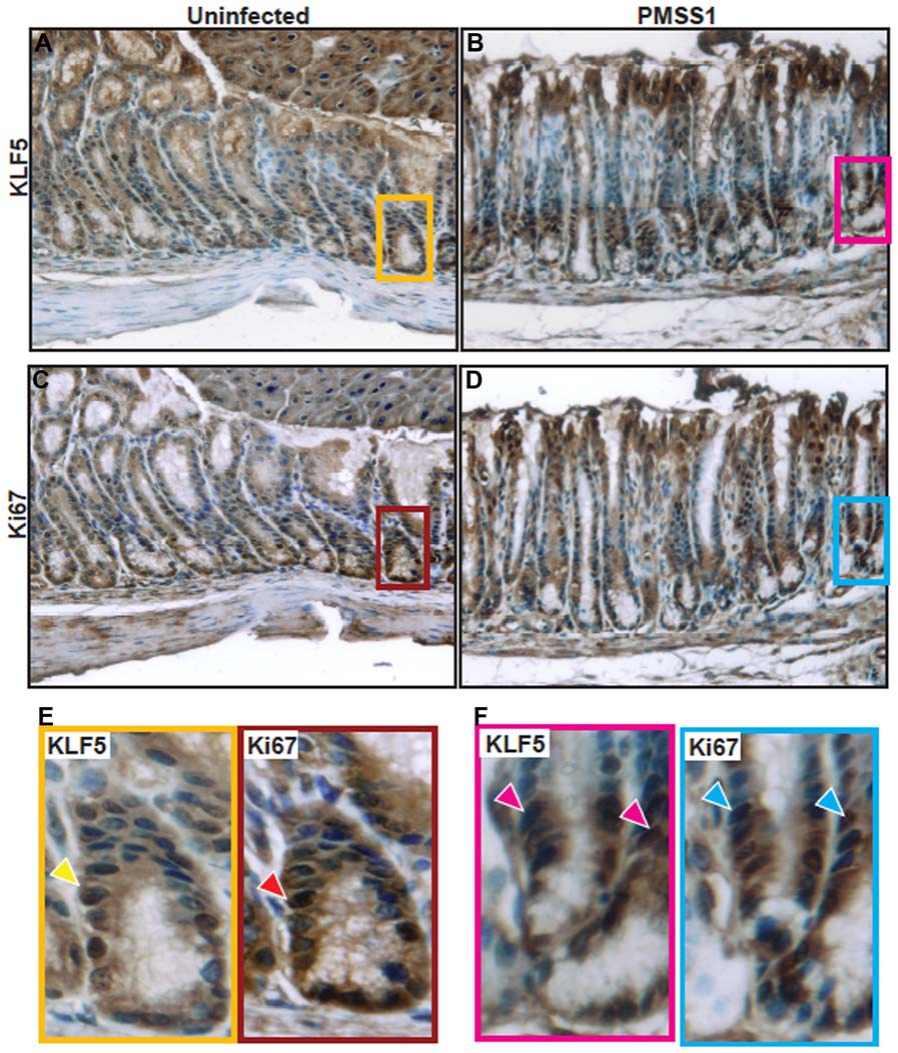
KLF5 and Ki67 co-localize to the isthmal region. KLF5 and Ki67 immunohistochemistry staining was assessed on murine gastric tissue sections from uninfected mice (A and C) or *H. pylori* PMSS1-infected mice (B and D) at 400× magnification. Insets demonstrate regions of KLF5 and Ki67 co-localization (arrows) within the isthmal regions of the gastric epithelium (E and F). Nuclei are stained in blue.

To further investigate the relationship between KLF5 expression and progenitor cell properties, we performed immunohistochemistry for Ki67 and KLF5 to highlight the isthmal region where stem cells are known to be located (Figure 7). These data demonstrate that both KLF5 and Ki67 co-localize to the isthmal region in uninfected (Figure 7A, 7C, and 7E) and *H. pylori-*infected (Figure 7B, 7D, and 7F) tissue sections and that this region is expanded upon infection.

### Human KLF5 expression increases in parallel with the severity of gastric neoplastic progression

**Figure 8:**
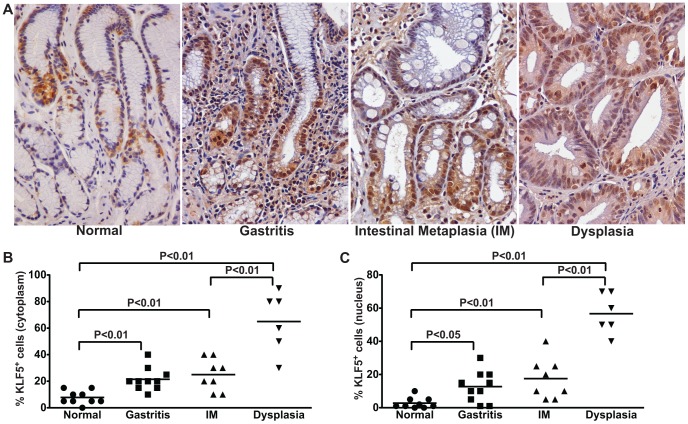
KLF5 expression parallels the severity of gastric premalignant lesions in *H. pylori*-infected humans. (A) KLF5 expression was evaluated by immunohistochemistry in a human population at high risk for gastric cancer. Gastric biopsies from uninfected patients with normal gastric mucosa and *H. pylori*-infected patients with non-atrophic gastritis, intestinal metaplasia (IM), and dysplasia were evaluated for KLF5 immunostaining at 200× magnification. (B and C) A single pathologist assessed the percentage of KLF5^+^ cells exhibiting cytoplasmic (B) or nuclear (C) staining. Each data point represents an individual biopsy and mean values are shown. The percentage and mean value of KLF5^+^ cells from biopsies from patients with normal gastric tissue (circles), gastritis (squares), intestinal metaplasia (IM, triangles), and dysplasia (inverted triangles) are shown. Mann-Whitney and ANOVA tests were used to determine statistical significance between groups.

To extend these findings into the natural niche of *H. pylori*, KLF5 expression was assessed by immunohistochemistry in *H. pylori*-negative individuals with normal gastric mucosa and *H. pylori*-infected subjects with non-atrophic gastritis, intestinal metaplasia (IM), or dysplasia. KLF5 expression paralleled the severity of gastric preneoplastic lesions (Figure 8A), such that there was a progressive increase in cytoplasmic (Figure 8B) and nuclear (Figure 8C) KLF5 immunostaining in foci of gastritis, intestinal metaplasia (IM), and dysplasia compared to normal gastric mucosa, and these increases were markedly augmented in patients with dysplasia. These data parallel our findings in an *in vitro* cell culture model as well as an *in vivo* murine model of *H. pylori* infection.

## Discussion

Krüppel-like factors (KLFs) function in the physiology and pathophysiology of several organ systems and many KLFs are involved in tumor biology [29], [30], [31], [32]. Expression of Krüppel-like factors is variable and cell- and tissue-specific; however, KLF5 expression is robust within the gastrointestinal tract, where it functions predominantly as a transcriptional activator [8], [9], [33].

The current data demonstrate that KLF5 is upregulated in gastric epithelial cells *in vitro* and *in vivo* following infection with *H. pylori*. Of interest, the *cag* type IV secretion system or its effector substrates CagA or peptidoglycan do not mediate *H. pylori*-induced KLF5 upregulation. Other known *H. pylori* virulence factors such as VacA are also not involved in the *H. pylori*-induced upregulation of KLF5. KLF5 expression is not dependent upon an active interplay with viable bacteria but does require direct contact with gastric epithelial cells, suggesting that upregulation of KLF5 is induced by a cell surface-exposed bacterial factor. Previous data have demonstrated that lipopolysaccharide (LPS), a bacterial-derived endotoxin, induces KLF5 expression in human cells [34]; however, our current data demonstrate that purified *H. pylori* LPS does not induce KLF5 expression in this *in vitro* cell culture model. We speculate, based on our results using heat-killed bacteria, that an outer membrane protein or proteins mediate *H. pylori*-induced upregulation of KLF5 and defining this factor will be an active focus of future studies.

KLF5 can function as a tumor suppressor or a tumor promoter, depending on the cell- and tissue-specific context. KLF5 expression is lost in breast cancer specimens, indicating a potential tumor suppressive role [35]. Conversely, several studies have demonstrated that KLF5 promotes breast cancer cell proliferation and survival [36], [37] and KLF5 expression has been associated with decreased breast cancer survival rates [38]. Overexpression of KLF5 in prostate cancer cell lines inhibits proliferation [39], [40]; however, *KLF5* transcript levels are consistently increased in prostate cancer samples, relative to normal prostate epithelium [41]. We have shown that KLF5 expression significantly increases in concordance with the severity of gastric premalignant lesions. Our data also demonstrate the involvement of *H. pylori* in the initial induction of KLF5 in gastric epithelial cells, which may contribute in part to sustained KLF5 expression throughout multiple stages of gastric neoplastic progression. However, the role of *H. pylori* and KLF5 at each of these stages warrants further investigation.

In addition to their role in tumor biology, KLFs have been shown to be involved in reprogramming somatic cells into inducible pluripotent stem cells and maintaining the pluripotent state of embryonic stem cells [42], [43], [44], [45]. Specifically, KLF5 is involved in embryonic stem cell self-renewal, maintenance, and proliferation in mice [46], [47]. We have now shown that, within the gastric epithelium of mice, *H. pylori* induces expansion of a murine KLF5^+^, Lrig1^+^ cell population. Lrig1 is expressed in gastric epithelial cells [28] and is enriched at the crypt base, specifically in the progenitor compartment of the small intestine and colon [48]. In addition to its expression in normal gastrointestinal tissues, Lrig1 is expressed at low levels in several types of cancer and is overexpressed in both prostate and colorectal tumors [49]. Our findings indicate that infection with *H. pylori* increases populations of murine gastric epithelial cells expressing both KLF5 and Lrig1, proteins implicated in tumor biology and stemness. Lrig1 acts to restrict stem cell proliferation [24], [48], a similar function ascribed to KLF5; thus, it is intriguing to speculate that these two elements may work in concert within *H. pylori*-infected gastric epithelium. Future work will focus on why this population of cells, normally restricted to the isthmal progenitor region, expands during *H. pylori* infection. Such lines of investigation may help define the contribution of these proteins to gastric carcinogenesis and maintenance of the gastric stem cell niche.

In conclusion, our results indicate that *H. pylori* can induce expression of KLF5 in gastric epithelial cells *in vitro* and *in vivo*. KLF5 expression increases in parallel with increasing severity of histologic lesions that comprise the cascade to gastric adenocarcinoma, which may provide insights into oncogenic events that develop in response to *H. pylori* infection.
